# Supplementary material for: Magnetic quasi-atomic electrons driven reversible structural and magnetic transitions between electride and its hydrides
Source: Nat Commun. 2023 Sep 6;14:5469. doi: 10.1038/s41467-023-41085-0 (PMC10482852; doi:10.1038/s41467-023-41085-0)
Supplement: Supplementary file 1 — Supplementary Information [file 41467_2023_41085_MOESM1_ESM.pdf]

Supplementary information

Magnetic quasi-atomic electrons driven reversible structural and magnetic transitions between electride and its hydrides

Seung Yong Lee[1,2,†], Dong Cheol Lim[1,3,†], Md Salman Khan[1,3,†], Jeong Yun Hwang[4], Hyung Sub Kim[5], Kyu Hyung Lee[4]*, Sung Wng Kim[1,3]*

[1]Department of Energy Science, Sungkyunkwan University, Suwon 16419, Republic of Korea.

[2]KIURI Institute, Yonsei University, Seoul 03722, Republic of Korea.

[3]Center for Electride Materials, Sungkyunkwan University, Suwon 16419, Republic of Korea.

[4]Department of Materials Science and Engineering, Yonsei University, Seoul 03722, Republic of Korea.

[5]Neutron Science Division, Korea Atomic Energy Research Institute, Daejeon 34057, Republic of Korea.

[†]These authors contributed equally to this work.

*e-mail: khlee2018@yonsei.ac.kr (K.H.L.) and kimsungwng@skku.edu (S.W.K.)

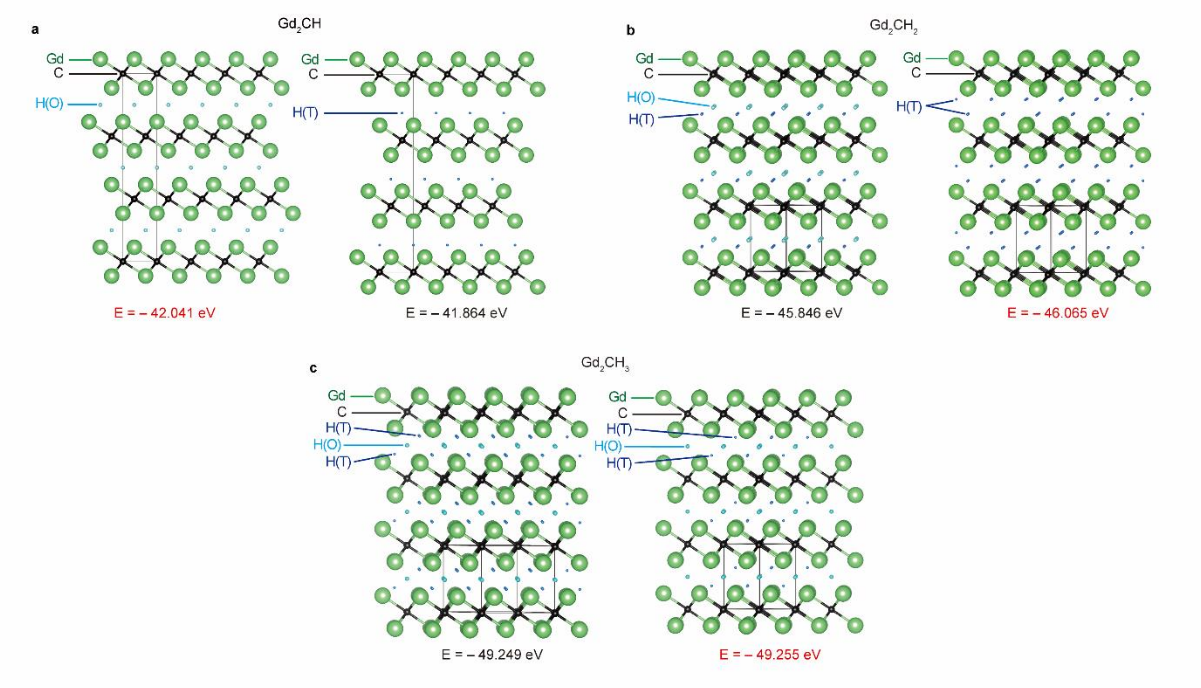

Supplementary Fig. 1 | Total energy calculations of $[Gd_2C]^{2+}\cdot 2e^{-}$ electride and its hydrides. Total energy comparison depending on the hydrogen concentration and position of Gd_2CH (**a**), Gd_2CH_2 (**b**), and Gd_2CH_3 (**c**), where the green, black, light blue and dark blue balls represent Gd, C, H(O), and H(T), respectively. Gd_2CH with hydrogen occupation at octahedral sites is preferred (left). Gd_2CH_2 with the hydrogen occupancy at the only tetrahedral sites is preferred (right). Gd_2CH_3 with hydrogen occupancy at both octahedral and tetrahedral sites is preferred to be crystallized into the $P\bar{3}1m$ structure (right). Left structure in **c** is crystallized in the $P\bar{3}m1$ structure.

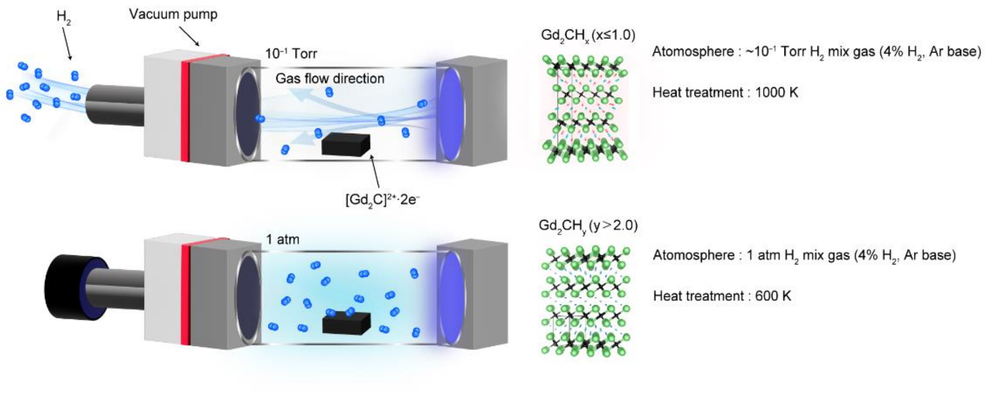

Supplementary Fig. 2 | Schematic illustration of synthetic process for the hydrogenation yielding Gd_2CH_x (x≤1.0) (top) and Gd_2CH_y (y>2.0) (bottom). The hydrogen pressures for each process producing hydrogenated Gd_2CH_x (x≤1.0) and Gd_2CH_y (y>2.0) were controlled with a vacuum pump station.

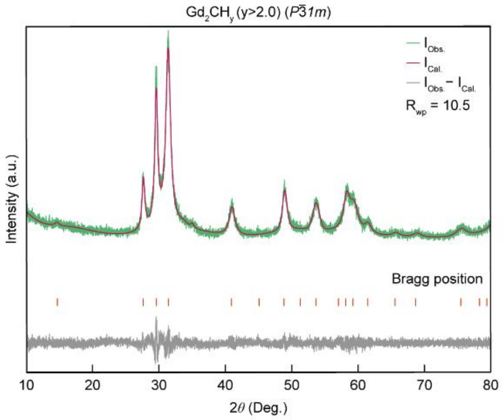

Supplementary Fig. 3 | Rietveld refinement of powder XRD pattern of Gd_2CH_y (y>2.0).

The structural parameters obtained by the refinement with the $P\bar{3}m1$ space group are listed in Table S1.

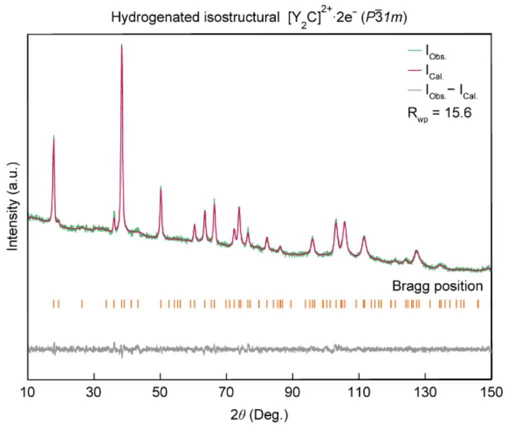

Supplementary Fig. 4 | Rietveld refinement of powder ND pattern of hydrogenated Y_2CH_y (y>2.0). The $[Y_2C]^{2+}\cdot 2e^-$ electride, which is isostructural with the $[Gd_2C]^{2+}\cdot 2e^-$ electride, was hydrogenated with the same condition to produce Gd_2CH_y (y>2.0) as shown in bottom of Supplementary Fig. 2.

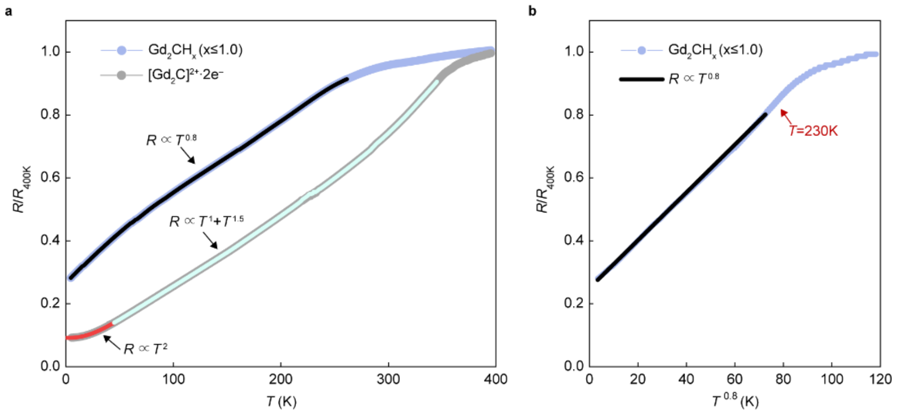

Supplementary Fig. 5 | Temperature dependence of electrical resistivity for the $[Gd_2C]^{2+}\cdot 2e^-$ electride and hydrogenated Gd_2CH_x (x≤1.0). a, Power law fitting of R/R_{400K} for metallic $[Gd_2C]^{2+}\cdot 2e^-$ electride and hydrogenated Gd_2CH_x (x≤1.0), showing different scattering mechanism of carriers. B, Plot of R/R_{400K} versus $T^{0.8}$ for the hydrogenated Gd_2CH_x (x≤1.0).

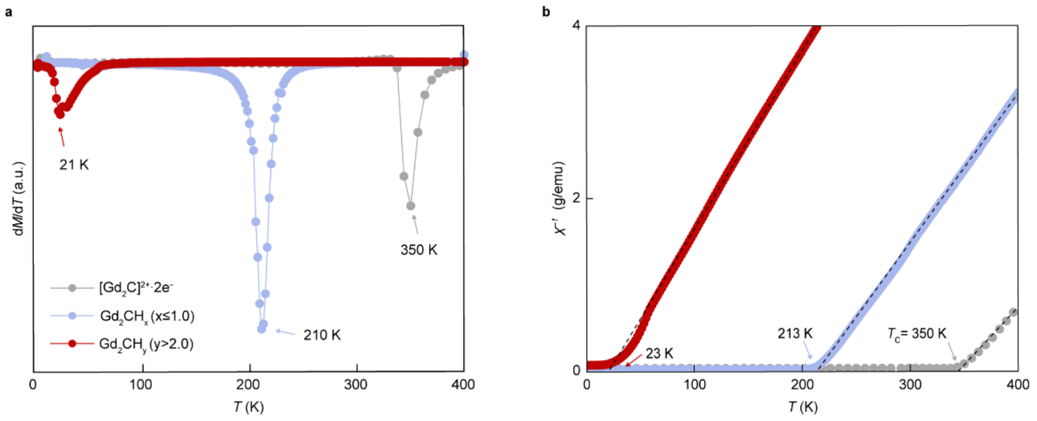

Supplementary Fig. 6 | Magnetic phase transition temperatures of the $[Gd_2C]^{2+}{\cdot}2e^{-}$ electride and its hydrides. A, Temperature dependence of d*M*/d*T* for the three samples. **B**, Curie-Weiss fitting for the three samples.

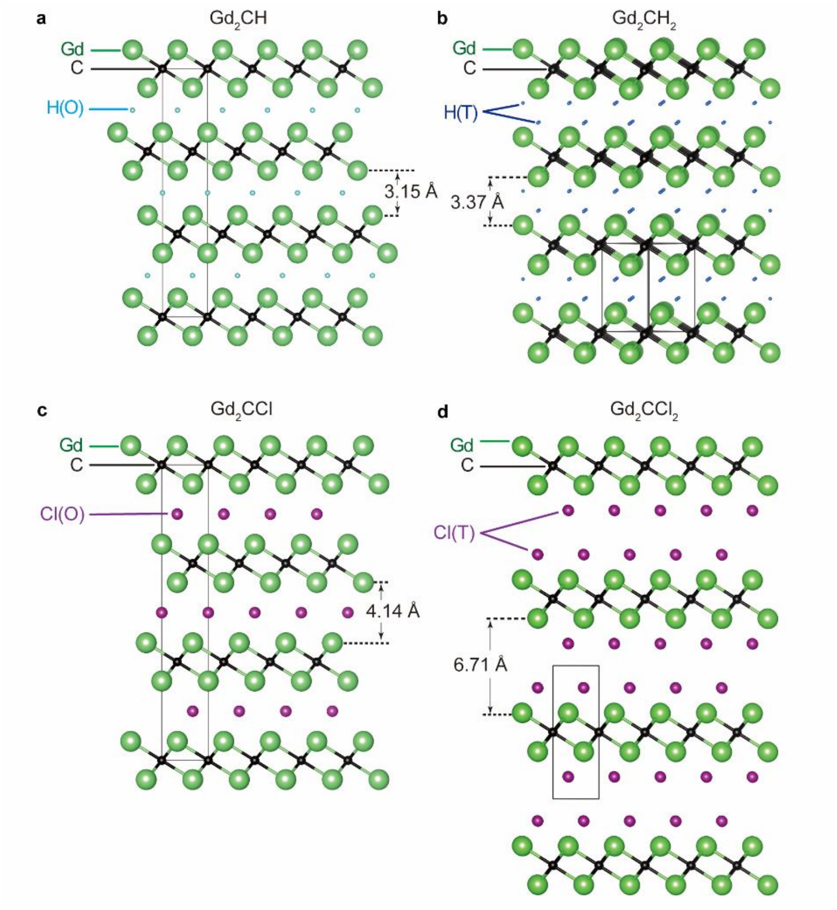

Supplementary Fig. 7 | Crystal structures of hydrogenated and halogenated Gd_2C compounds. Comparison of hydrogenated (Gd_2CH (**a**) and Gd_2CH_2 (**b**)) and halogenated (Gd_2CCl (**c**) and Gd_2CCl_2 (**d**)) compounds. Green, black, light blue, dark blue, and purple balls represent Gd, C, H(O), H(T), and Cl atoms, respectively.

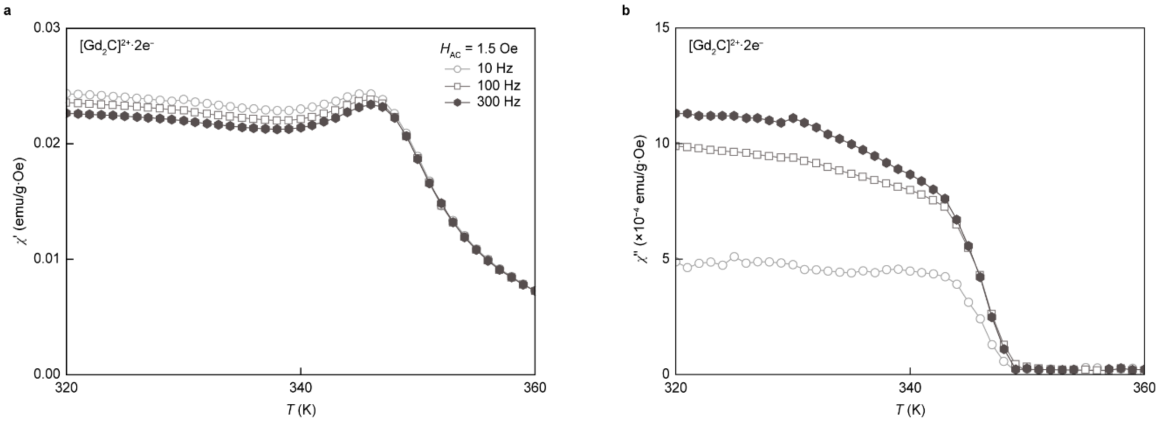

Supplementary Fig. 8 | AC susceptibility of ferromagnetic $[Gd_2C]^{2+}{\cdot}2e^-$ electride. Real part (**a**) and imaginary part (**b**) of AC susceptibility are measured under different frequencies at 0 H_{DC}.

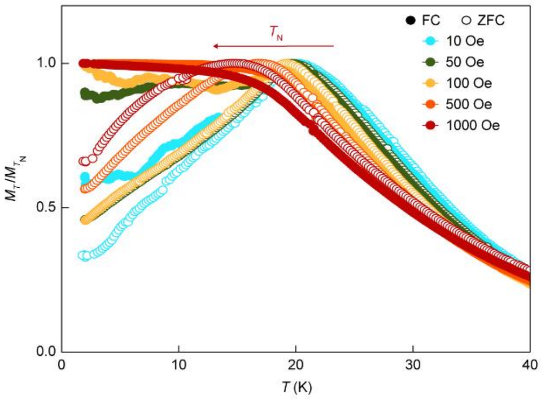

Supplementary Fig. 9 | Antiferromagnetic nature of hydrogenated Gd_2CH_y (y>2.0). The T_N decreasing with the increase of DC magnetic field is clearly revealed from M_T/M_{T_N}.

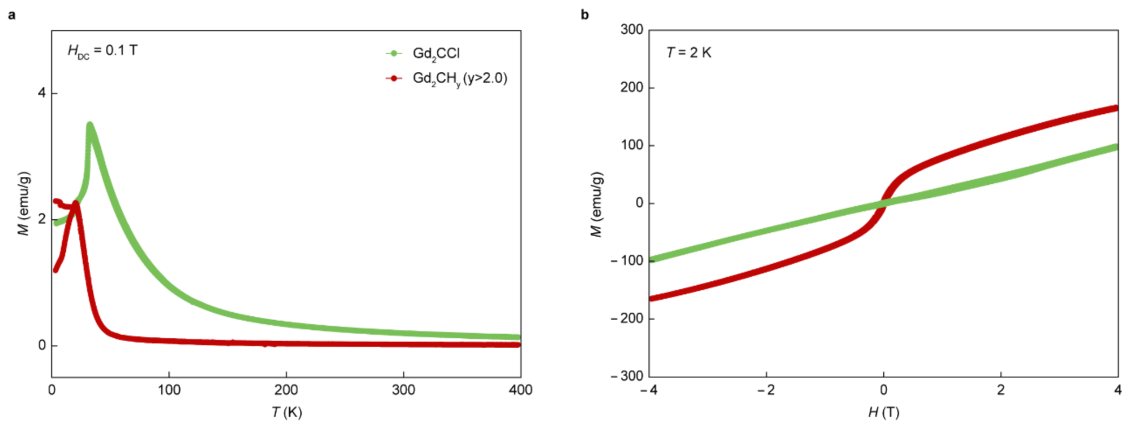

Supplementary Fig. 10 | Magnetic properties between Cl-substituted Gd_2CCl and H-substituted Gd_2CH_y (y>2.0). a, *M*–*T* curves measured under magnetic field of 0.1 T, showing no splitting and a clear splitting between ZFC and FC for Gd_2CCl and Gd_2CH_y (y>2.0), respectively. **b**, *M*–*H* curves measured at 2 K. These results strongly indicate the antiferromagnetism of Gd_2CCl and canted antiferromagnetsm of Gd_2CH_y (y>2.0).

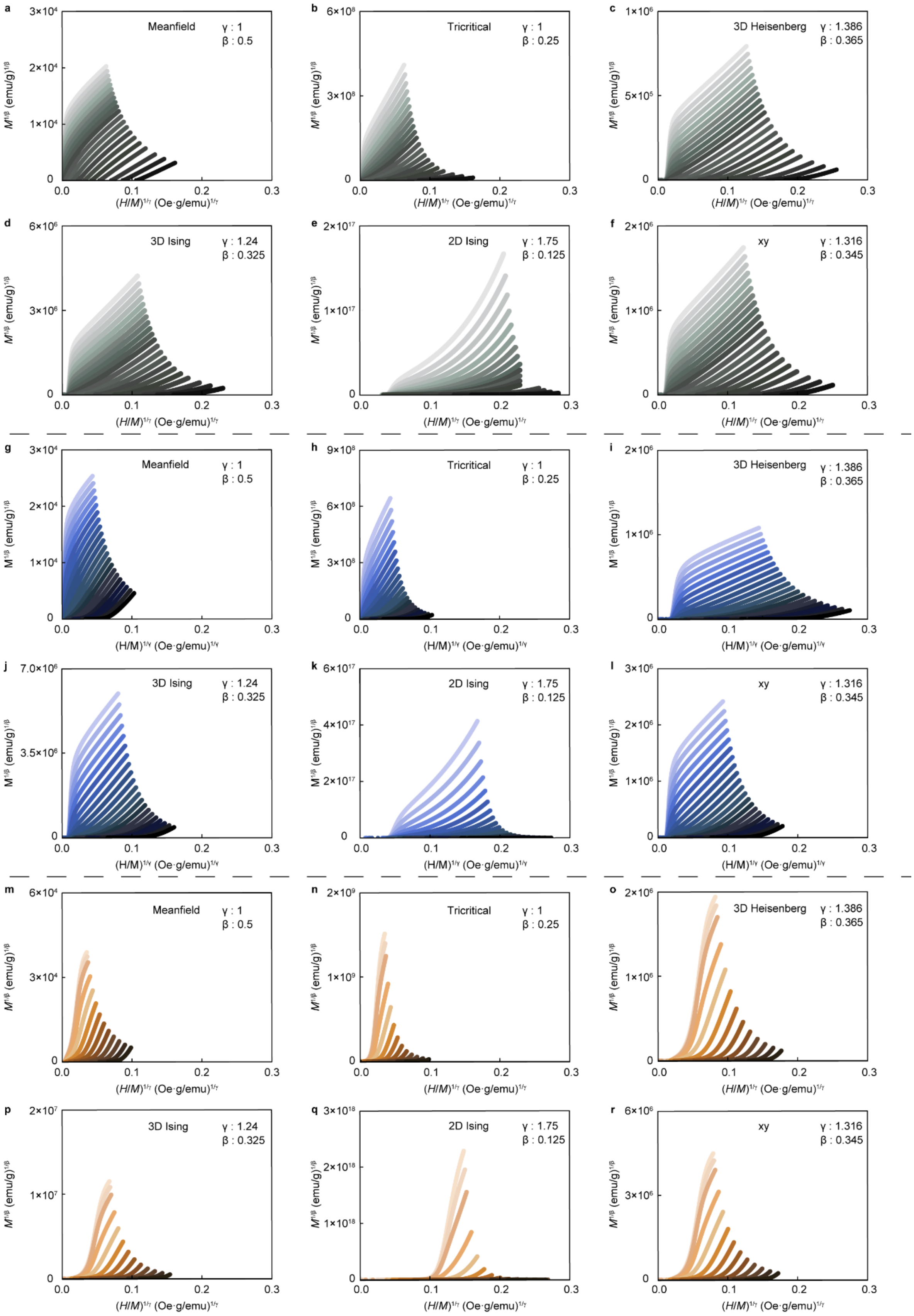

a
Meanfield
γ : 1
β : 0.5
3×10⁴
2×10⁴
1×10⁴
0
$M^{1/\beta}$ (emu/g)$^{1/\beta}$
0.0
0.1
0.2
0.3
$(H/M)^{1/\gamma}$ (Oe·g/emu)$^{1/\gamma}$
b
Tricritical
γ : 1
β : 0.25
6×10⁸
3×10⁸
0
c
3D Heisenberg
γ : 1.386
β : 0.365
1×10⁶
5×10⁵
0
d
3D Ising
γ : 1.24
β : 0.325
6×10⁶
3×10⁶
0
e
2D Ising
γ : 1.75
β : 0.125
2×10¹⁷
1×10¹⁷
0
f
xy
γ : 1.316
β : 0.345
2×10⁶
1×10⁶
0
g
Meanfield
γ : 1
β : 0.5
3×10⁴
2×10⁴
1×10⁴
0
h
Tricritical
γ : 1
β : 0.25
9×10⁸
6×10⁸
3×10⁸
0
i
3D Heisenberg
γ : 1.386
β : 0.365
2×10⁶
1×10⁶
0
j
3D Ising
γ : 1.24
β : 0.325
7.0×10⁶
3.5×10⁶
0
k
2D Ising
γ : 1.75
β : 0.125
6×10¹⁷
4×10¹⁷
2×10¹⁷
0
l
xy
γ : 1.316
β : 0.345
3×10⁶
2×10⁶
1×10⁶
0
m
Meanfield
γ : 1
β : 0.5
6×10⁴
3×10⁴
0
n
Tricritical
γ : 1
β : 0.25
2×10⁹
1×10⁹
0
o
3D Heisenberg
γ : 1.386
β : 0.365
2×10⁶
1×10⁶
0
p
3D Ising
γ : 1.24
β : 0.325
2×10⁷
1×10⁷
0
q
2D Ising
γ : 1.75
β : 0.125
3×10¹⁸
2×10¹⁸
1×10¹⁸
0
r
xy
γ : 1.316
β : 0.345
6×10⁶
3×10⁶
0

Supplementary Fig. 11 | The modified Arrott plot of $[Gd_2C]^{2+}\cdot 2e^{-}$ electride and its hydrides. a–f, Modified Arrott plots of $[Gd_2C]^{2+}\cdot 2e^{-}$ electride with meanfield (**a**), tricritical (**b**), 3D Heisenberg (**c**), 3D Ising (**d**), 2D Ising (**e**), xy (**f**) models. **g–l**, Modified Arrott plots **of** Gd_2CH_x (x≤1.0) with meanfield (**g**), tricritical (**h**), 3D Heisenberg (**i**), 3D Ising (**j**), 2D Ising (**k**), xy (**l**) models. **m–r**, Modified Arrott plots **of** Gd_2CH_y (y>2.0) with meanfield (**m**), tricritical (**n**), 3D Heisenberg (**o**), 3D Ising (**p**), 2D Ising (**q**), xy (**r**) models.

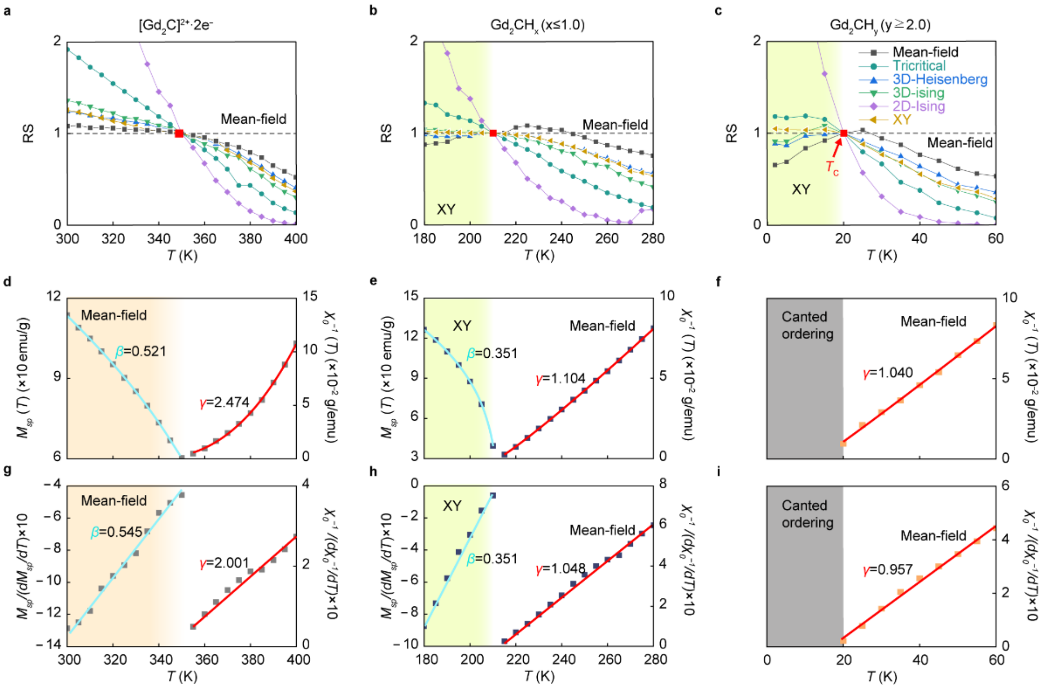

Supplementary Fig. 12 | Critical exponent fitting from Arrott plot. a–c, Temperature dependence of relative slope (RS) curves near the magnetic transition temperatures for the $[Gd_2C]^{2+}\cdot 2e^-$ electride (a), Gd_2CH_x ($x \leq 1.0$) (**b**) and Gd_2CH_y ($y > 2.0$) (**c**). **d–f**, Plots of the critical exponent model calculated by M_S and χ_0^{-1} from Arrott plots for each sample. **g–i**, Critical exponent value derives from KF method.

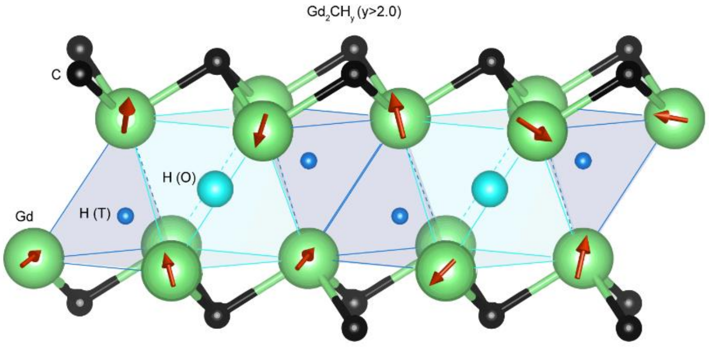

Supplementary Fig. 13 | Schematic illustration of canted antiferromagnetic ordering in Gd_2CH_y (y>2.0). Out-of-plane interaction between Gd–H–Gd determines the canted antiferromagnetism.

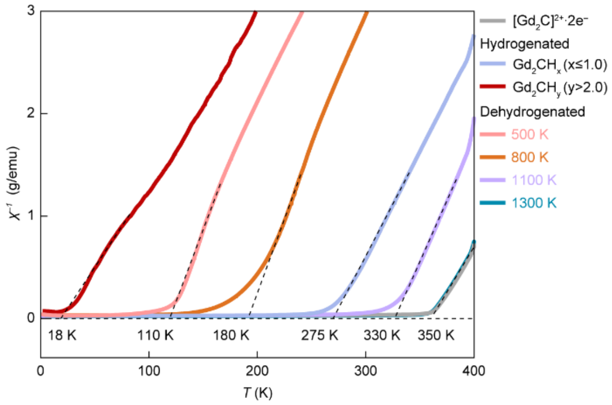

Supplementary Fig. 14 | Curie-Weiss fitting. Temperature dependence of reciprocal magnetic susceptibility (χ^{-1}) of $[Gd_2C]^{2+}\cdot 2e^{-}$ electride, hydrogenated and dehydrogenated samples.

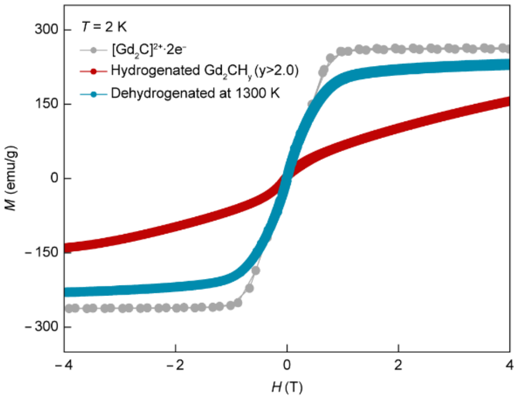

Supplementary Fig. 15 | *M*–*H* curves of the $[Gd_2C]^{2+}·2e^{-}$ electride and its hydrides. The data was obtained at 2 K.

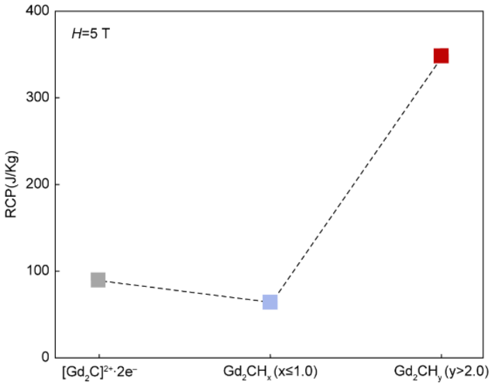

Supplementary Fig. 16 | RCP performance of the $[Gd_2C]^{2+}\cdot 2e^{-}$ electride and its hydrides. The canted antiferromagnetic Gd_2CH_y (y>2.0) shows a larger RCP value than those of ferromagnetic $[Gd_2C]^{2+}\cdot 2e^{-}$ electride and Gd_2CH_x (x≤1.0) samples.

Supplementary Table 1 | Crystal structure and structural parameters of Gd_2CH and Gd_2CCl.

Sample	Gd_2CH	Gd_2CCl
Space group	$R\bar{3}m$	$R\bar{3}m$
a, b (Å)	3.64	3.69
c (Å)	18.13	20.35
Volume (Å^3)	207.55	240.24

Atom	Position / Occupancy	
Gd1	(0 0 0.257) / 1.0	(0 0 0.268) / 1.0
Gd2		
C1	(0 0 0) / 1.0	(0 0 0) / 1.0
C2	(0.333 0.667 0) / 1.0	(0.333 0.667 0) / 1.0
H/Cl	(0 0 0.5)	(0 0 0.5)

Supplementary Table 2 | XRD Rietveld results of Gd_2CH_x (x≤1.0) and Gd_2CH_y (y>2.0).

Sample	Gd_2CH_x (x≤1.0)	Gd_2CH_y (y>2.0)		
**Space group**	$R\bar{3}m$	$P\bar{3}1m$	$P\bar{3}m1$	$P\bar{3}m1$ *(Supplementary Fig. 2)*
**a, b (Å)**	3.64	6.47	3.74	3.73
**c (Å)**	18.13	6.02	6.02	6.03
**Volume (Å^3)**	207.55	218.79	72.88	72.59

Atom	Position / Occupancy			
**Gd1**	(0 0 0.257) / 1.0	(0.350 0 0.247) / 1.0	(0.333 0.667 0.269) / 1.0	(0.667 0.333 0.548) / 1.0
**Gd2**				(0 0 0) / 1.0
**C1**	(0 0 0) / 1.0	(0 0 0) / 1.0	(0 0 0) / 1.0	(0.333 0.667 0.77) / 1.0
**C2**	(0.333 0.667 0) / 1.0	(0.333 0.667 0) / 1.0		
R_{wp} (%)	8.71	8.17	8.80	10.50

Supplementary Table 3 | ND Rietveld results of hydrogenated $[Y_2C]^{2+}\cdot 2e^-$.

Space group	$P\bar{3}1m$
**a, b (Å)**	6.34
**c (Å)**	5.94
**Volume (Å^3)**	206.695

Atom	Position / Occupancy
**Y**	(0.345 0 0.250) / 1.0
**C1**	(0 0 0) / 1.0
**C2**	(0.333 0.667 0) / 1.0
**H1**	(0 0 0.500) / 1.0
**H2**	(0.333 0.667 0.500) / 0.62
**H3**	(0.298 0 0.625) / 0.79
R_{wp} (%)	15.6

Supplementary Table 4 | Crystal structure and structural parameters of Gd_2CH_2 and Gd_2CCl_2.

Sample	Gd_2CH_2	Gd_2CCl_2
**Space group**	$P\bar{3}m1$	$P\bar{3}m1$
**a, b (Å)**	3.72	3.76
**c (Å)**	6.12	9.46
**Volume (Å^3)**	73.42	116.02

Atom	Position / Occupancy	
**Gd1**	(0 0 0.257) / 1.0	(0.333 0.667 0.355) / 1.0
**Gd2**		
**C1**	(0 0 0)/ 1.0	(0 0 0.5) / 1.0
**C2**	(0.333 0.667 0) / 1.0	
**H/Cl**	(0 0 0.257)	(0.333 0.667 0.834)
